# Cryosurgery for the treatment of cutaneous sporotrichosis in four pregnant women

**DOI:** 10.1371/journal.pntd.0006434

**Published:** 2018-04-23

**Authors:** Vivian Fichman, Antonio Carlos Francesconi do Valle, Priscila Marques de Macedo, Dayvison Francis Saraiva Freitas, Manoel Marques Evangelista de Oliveira, Rodrigo Almeida-Paes, Maria Clara Gutierrez-Galhardo

**Affiliations:** 1 Laboratory of Clinical Research on Infectious Dermatology, Evandro Chagas National Institute of Infectious Diseases, Oswaldo Cruz Foundation (Fiocruz), Rio de Janeiro, Brazil; 2 Laboratory of Mycology, Evandro Chagas National Institute of Infectious Diseases, Oswaldo Cruz Foundation (Fiocruz), Rio de Janeiro, Brazil; University of Tennessee, UNITED STATES

## Abstract

**Background:**

Pregnant women with sporotrichosis should not receive systemic antifungal therapy except in severe cases when amphotericin B is recommended. Thermotherapy is the most reported treatment described in this group of patients. It entails weeks of daily self-application of heat to the lesions, requires that the patient faithfully apply it, and it could cause skin burns. Cryosurgery is a useful therapeutic tool for many cutaneous infectious diseases, safe for pregnant women, but not well evaluated for sporotrichosis treatment in this group.

**Methodology:**

The authors conducted a retrospective study describing epidemiological, clinical, and therapeutic data related to four pregnant patients with sporotrichosis treated with cryosurgery. The authors reviewed the clinical records of four pregnant patients diagnosed with cutaneous sporotrichosis and treated with cryosurgery. The sessions were carried out monthly up to clinical cure. Molecular identification of the *Sporothrix* species was performed in two cases using T3B PCR fingerprinting assays.

**Principal findings:**

All patients were in the second trimester of pregnancy and their age ranged from 18 to 34 years. With regard to clinical presentation, two patients had lymphocutaneous and two had the fixed form. *S*. *brasiliensis* was identified in two cases as the causative agent. Cryosurgery was well tolerated and the number of sessions ranged from 1 to 3. All the patients reached a complete clinical cure.

**Conclusions:**

Cryosurgery was a safe, easy to perform and well tolerated method, and therefore it is suggested to be a suitable option for the treatment of cutaneous sporotrichosis in pregnant women.

## Author summary

Sporotrichosis is a cosmopolitan disease, considered the most important subcutaneous mycosis in Latin America. Since 1998, there is an ongoing cat-transmitted zoonotic epidemic of sporotrichosis occurring in Rio de Janeiro, Brazil. Pregnant women are a vulnerable population occasionally affected that require special attention regarding sporotrichosis treatment. Antifungal drugs should be avoided because of their potential risks to the fetus, unless in severe cases when amphotericin B (an intravenous antifungal drug) can be indicated. In this context, local measures are the treatment of choice. Cryosurgery consists in local application of intense cold using liquid nitrogen to destroy some infectious, tumoral and inflammatory cutaneous diseases. It is scarcely reported in the literature for the treatment of sporotrichosis, especially in pregnant women for whom local heat is most used. This works aims to describe the clinical response and outcome of cryosurgery for the treatment of sporotrichosis in four pregnant women. All patients reached clinical cure after one to three sessions. These results suggest that cryosurgery can be a well-tolerated, safe, and efficient method for the treatment of sporotrichosis in pregnancy.

## Introduction

Sporotrichosis is caused by dimorphic fungi of the genus *Sporothrix*, found in its filamentous form as saprophytes on decaying and living vegetation, and soil [1]. However, since the late 1990s, sporotrichosis in the state of Rio de Janeiro, Brazil, has become an urban-epidemic phenomenon, being transmitted from naturally infected cats to humans [2]. The most affected population is characterized by having poor socioeconomic backgrounds and low access to health services. In this zoonotic scenario of sporotrichosis transmission, female patients with a median age of 39 years predominate, and most of them acquire the disease through bite or scratches from infected cats [2]. In this context, women in childbearing age are an at-risk population to acquire this mycosis.

Sporotrichosis in pregnancy is a therapeutic challenge. Pregnant women should not receive azole therapy due to the potential teratogenic effects, as well as potassium iodide saturated solution (SSKI), because of its toxicity to the fetal thyroid. Although terbinafine is classified by the US Food and Drug Administration (FDA) as a category B drug, there is no sufficient clinical experience in pregnancy. Besides that, terbinafine passes into the breast milk, which could have an effect on a nursing baby. For severe sporotrichosis cases that need to be treated during pregnancy, amphotericin B is recommended [3–5].

Since systemic treatment is hardly possible, local alternative treatment plays an important role in pregnancy. Thermotherapy is the most reported therapeutic option described in this group of patients entailing weeks of daily self-application of heat to the lesions, and requires a faithfully application with a certain caution to avoid skin burns [3–7]. Cryosurgery is an effective and safe method, when applied by well-trained staff, being a useful therapeutic resource for many infectious skin diseases [8, 9]. Regarding the treatment of cutaneous sporotrichosis, it has already been reported as an effective adjuvant therapy when associated with oral antifungals [9–11]. However, to the best of our knowledge, has not yet been evaluated in pregnant women. The authors report four cases of pregnant women with cutaneous sporotrichosis that were successfully treated with cryosurgery.

## Materials and methods

The study was approved by the Ethical Committee of the INI, Fiocruz, Rio de Janeiro, Brazil (CAAE 55348416.5.0000.5262). The patients’ data were anonymized/de-identified to protect patients’ privacy/confidentiality.

The authors reviewed the clinical records of pregnant patients diagnosed with cutaneous sporotrichosis who were treated at the cryosurgery outpatient clinic of the Laboratory of Clinical Research in Infectious Dermatology, Evandro Chagas National Institute of Infectious Diseases (INI), Oswaldo Cruz Foundation (Fiocruz) from 2006 to 2016. Briefly, the protocol of pregnant women with sporotrichosis included isolation of *Sporothrix* spp. in clinical specimens [2], complete blood count, and biochemical tests. They were instructed to perform thermotherapy with warm compresses for 20 minutes 3 times a day [7]. Subsequent follow-up was scheduled monthly or anytime in case of worsening of the lesions. For non-adherent patients or those who did not desire to perform thermotherapy for sporotrichosis treatment, cryosurgery was offered, and that was the case of the patients included in this work. Patients that received any other type of treatment for sporotrichosis besides cryosurgery were excluded. Cryosurgery sessions were carried out monthly, performed by dermatologists, up to clinical cure. In each session, lesions were treated with two cycles of 10 to 30 seconds of freeze time with liquid nitrogen in spray form. Clinical cure was defined as complete healing of the lesions. In a general way, once *Sporothrix* spp. was isolated, molecular identification of the species was performed using the T3B PCR fingerprinting method [12].

## Results

**Fig 1:**
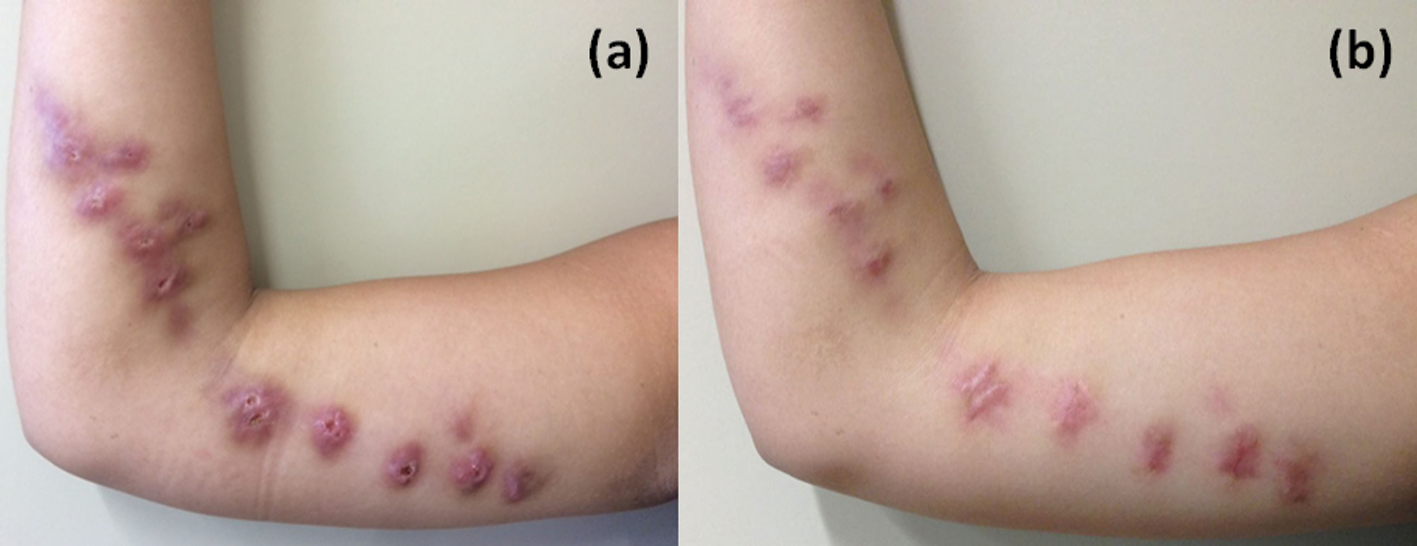
a) Case 2 presenting lymphocutaneous sporotrichosis in the arm; b) The same patient with complete healed lesions after 2 sessions of cryosurgery.

From 2006 to 2016, 218 adult patients diagnosed with sporotrichosis, by fungal isolation in culture, were treated with cryosurgery. From these 218 patients, 8 were pregnant women, and 4 of them were treated exclusively with cryosurgery. These 4 patients were at the second trimester of pregnancy and their age ranged from 18 to 34 years. All of them lived in Rio de Janeiro state, Brazil. Two of them worked with domestic duties. The patients presented ulcerovegetative or nodular ulcerovegetative lesions (Fig 1).

Due to technical reasons, molecular identification of the agent was feasible in two patients (cases 3 and 4—Table 1), and the isolates were identified as *S*. *brasiliensis*. Complete blood count, and biochemical tests performed before, during, and after the treatment were within the normal ranges. Cryosurgery was well tolerated with no need for local anesthesia. The number of cryosurgery sessions ranged from 1 to 3. All the patients were discharged after a complete cure. No adverse reactions were observed during the treatment as well as no relapses were documented after delivery. Epidemiological, clinical, and therapeutic data of the patients are detailed in Table 1.

**Table 1:** Epidemiological, clinical and therapeutic aspects of four pregnant patients with sporotrichosis treated with cryosurgery.

Case	Age (years)	Gestational age (weeks)	Transmission form	Clinical presentation	Lesion Sites	Number of cryosurgery sessions
1	18	20	Contact with infected cat	Fixed cutaneous Ulcerovegetative	Thigh	2
2	22	16	Unknown	Lymphocutaneous Nodular ulcerative	Arm	2
3	34	20	Contact with cat	Fixed cutaneous Ulcerovegetative	Shoulder	1
4	32	24	Scratch from infected cat	Lymphocutaneous Ulcero vegetative and nodular lesions	Hand and forearm	3

## Discussion

Despite the low number of pregnant women affected and the benign clinical course, the treatment of sporotrichosis in pregnancy can be always considered challenging. In these cases, topical alternative therapeutic resources are safer than systemic drugs and should be considered whenever possible. Since the 1950s, thermotherapy was reported as the unique topical method for the treatment of sporotrichosis in pregnancy, with a strength of recommendation and quality of evidence considered as BIII [4]. Cryosurgery emerges as a useful tool for many infectious skin diseases, with effects of local cellular and humoral inflammatory response induction in the tissue, with its necrotic effect and, consequently, destructive for the infectious agents [8, 9].

Cryosurgery has been used as an adjuvant treatment in sporotrichosis, especially in residual lesions or in cases of ulcerovegetative or nodular ulcerovegetative thick lesions since it allows a good penetration of liquid nitrogen in spray form [13]. In other subcutaneous mycoses such as chromoblastomycosis, cryosurgery has been indicated as an isolated method or associated to systemic antifungal agents with good results [14]. Some authors have warned about the risk of lymphatic dissemination with invasive methods performed without systemic drugs in cases of chromoblastomycosis [15, 16]. In contrast with other procedures, cryosurgery is not only an ablative technique but also promotes an immune response, what could reduce this risk. A recent study with murine model found that cryosurgery was responsible for an increase in antigen-presenting dendritic cells (DCs), neutrophils and macrophages in subcutaneous tissue, as well as migration of DCs to regional lymph nodes [17]. Cryosurgery is contraindicated for patients who are sensitive to cold (cold urticaria, cryoglobulinemia, or cryofibrinogenemia) and should be avoided in extensive lesions or flexor surfaces due to the risk of fibrosis [18]. Until now, cryosurgery for sporotrichosis treatment has been poorly explored and documented especially considering cases that involve a supposed more virulent phylogenetic species such as *S*. *brasiliensis*.

All patients herein reported came from hyperendemic areas of sporotrichosis in Rio de Janeiro state, and become infected during pregnancy. None referred prior trauma with plants, but only contact and/or trauma with cats, in agreement with the zoonotic epidemic profile reported in the literature [2]. Although *S*. *brasiliensis*, could be identified in only two cases, it is well known that it is the main species involved in Rio de Janeiro epidemic. All patients presented cutaneous-limited clinical forms on the extremities, similar to previous publications [3, 5], in contrast with other mycoses, which can be more aggressive during pregnancy [19]. This work suggests that cryosurgery is a safe and well-tolerated method, easy to perform, being a promising alternative in the treatment of cutaneous sporotrichosis in pregnant women. Further studies with a larger number of patients are necessary to confirm efficacy of cryosurgery for sporotrichosis in pregnant patients.

## Supporting information

S1 Checklist STROBE checklist. (DOC)
