# Supplementary material for: Cryosurgery for the treatment of cutaneous sporotrichosis in four pregnant women
Source: PLoS Negl Trop Dis. 2018 Apr 23;12(4):e0006434. doi: 10.1371/journal.pntd.0006434 (PMC5933803; doi:10.1371/journal.pntd.0006434)
Supplement: S1 Checklist — (DOC) [file pntd.0006434.s001.doc]

STROBE Statement—Checklist of items that should be included in reports of ***observational studies***

|  | Item No | Recommendation |
| --- | --- | --- |
| **Title and abstract** | 1 | (*a*) Indicate the study’s design with a commonly used term in the title or the abstract  Lines 38-40 (abstract): “The authors conducted a retrospective study describing epidemiological, clinical, and therapeutic data related to four pregnant patients with sporotrichosis treated with cryosurgery.” |
|  |  | (*b*) Provide in the abstract an informative and balanced summary of what was done and what was found – Lines 38 to 51: The abstract includes methodology description, summarized results data and a final conclusion. “Methodology: The authors conducted a retrospective study describing epidemiological, clinical, and therapeutic data related to four pregnant patients with sporotrichosis treated with cryosurgery. The authors reviewed the clinical records of four pregnant patients diagnosed with cutaneous sporotrichosis and treated with cryosurgery. The sessions were carried out monthly up to clinical cure. Molecular identification of the Sporothrix species was performed in two cases using T3B PCR fingerprinting assays. Principal Findings: All patients were in the second trimester of pregnancy and their age ranged from 18 to 34 years. With regard to clinical presentation, two patients had lymphocutaneous and two had the fixed form. *S. brasiliensis* was identified in two cases as the causative agent. Cryosurgery was well tolerated and the number of sessions ranged from 1 to 3. All the patients reached a complete clinical cure. Conclusions: Cryosurgery was a safe, easy to perform and well tolerated method, and therefore it is suggested to be a suitable option for the treatment of cutaneous sporotrichosis in pregnant women.” |
| Introduction | | |
| Background/rationale | 2 | Explain the scientific background and rationale for the investigation being reported  Lines 76 to 95 – “Sporotrichosis is caused by dimorphic fungi of the genus *Sporothrix*, found in its filamentous form as saprophytes on decaying and living vegetation, and soil [1]. However, since the late 1990s, sporotrichosis in the state of Rio de Janeiro, Brazil, has become an urban-epidemic phenomenon, being transmitted from naturally infected cats to humans [2]. The most affected population is characterized by having poor socioeconomic backgrounds and low access to health services. In this zoonotic scenario of sporotrichosis transmission, female patients with a median age of 39 years predominate, and most of them acquire the disease through bite or scratches from infected cats [2]. In this context, women in childbearing age are an at-risk population to acquire this mycosis.  Sporotrichosis in pregnancy is a therapeutic challenge. Pregnant women should not receive azole therapy due to the potential teratogenic effects, as well as potassium iodide saturated solution (SSKI), because of its toxicity to the fetal thyroid. Although terbinafine is classified by the US Food and Drug Administration (FDA) as a category B drug, there is no sufficient clinical experience in pregnancy. Besides that, terbinafine passes into the breast milk, which could have an effect on a nursing baby. For severe sporotrichosis cases that need to be treated during pregnancy, amphotericin B is recommended [3-5].  Since systemic treatment is hardly possible, local alternative treatment plays an important role in pregnancy. Thermotherapy is the most reported therapeutic option described in this group of patients entailing weeks of daily self-application of heat to the lesions, and requires a faithfully application with a certain caution to avoid skin burns [3-5, 7]. Cryosurgery is an effective and safe method, when applied by well-trained staff, being a useful therapeutic resource for many infectious skin diseases [8, 9]. Regarding the treatment of cutaneous sporotrichosis, it has already been reported as an effective adjuvant therapy when associated with oral antifungals [9-11]. However, to the best of our knowledge, has not yet been evaluated in pregnant women.” |
| Objectives | 3 | State specific objectives, including any prespecified hypotheses  Lines 95 to 97 – “The authors report four cases of pregnant women with cutaneous sporotrichosis that were successfully treated with cryosurgery.” |
| Methods | | |
| Study design | 4 | Present key elements of study design early in the paper  Lines 103 to 107 “The authors reviewed the clinical records of pregnant patients diagnosed with cutaneous sporotrichosis who were treated at the cryosurgery outpatient clinic of the Laboratory of Clinical Research in Infectious Dermatology, Evandro Chagas National Institute of Infectious Diseases (INI), Oswaldo Cruz Foundation (Fiocruz) from 2006 to 2016.” |
| Setting | 5 | Describe the setting, locations, and relevant dates, including periods of recruitment, exposure, follow-up, and data collection  Lines 104 to 107– “treated at the cryosurgery outpatient clinic of the Laboratory of Clinical Research in Infectious Dermatology, Evandro Chagas National Institute of Infectious Diseases (INI), Oswaldo Cruz Foundation (Fiocruz) from 2006 to 2016.” |
| Participants | 6 | (*a*) Give the eligibility criteria, and the sources and methods of selection of participants. Describe methods of follow-up.  Lines 107 to 117 – “Brieﬂy, the protocol of pregnant women with sporotrichosis included isolation of *Sporothrix* spp. in clinical specimens [2], complete blood count, and biochemical tests. They were instructed to perform thermotherapy with warm compresses for 20 minutes 3 times a day [7]. Subsequent follow-up was scheduled monthly or anytime in case of worsening of the lesions. For non-adherent patients or those who did not desire to perform thermotherapy for sporotrichosis treatment, cryosurgery was offered, and that was the case of the patients included in this work. Patients that received any other type of treatment for sporotrichosis besides cryosurgery were excluded. Cryosurgery sessions were carried out monthly, performed by dermatologists, up to clinical cure. In each session, lesions were treated with two cycles of 10 to 30 seconds of freeze time with liquid nitrogen in spray form. Clinical cure was defined as complete healing of the lesions.” |
|  |  | (*b*) For matched studies, give matching criteria and number of exposed and unexposed – Not applicable. |
| Variables | 7 | Clearly define all outcomes, exposures, predictors, potential confounders, and effect modifiers. Give diagnostic criteria, if applicable  Lines 107 to 108: “included isolation of *Sporothrix* spp. in clinical specimens [2], complete blood count, and biochemical tests.” |
| Data sources/ measurement | 8* | For each variable of interest, give sources of data and details of methods of assessment (measurement). Describe comparability of assessment methods if there is more than one group.  Only one group is applicable. Variables of interest (epidemiological, clinical, prognostic and laboratory) were obtained from medical records and the method of measurement is detailed. Lines 117 to 119. |
| Bias | 9 | Describe any efforts to address potential sources of bias.  It is a descriptive study including all patients that fulfilled the inclusion criteria of the selected period and whose evaluation was standardized based on an international consensus. Lines 113 and 114: “ Patients that received any other type of treatment for sporotrichosis besides cryosurgery were excluded.” |
| Study size | 10 | Explain how the study size was arrived at. Not applicable. |
| Quantitative variables | 11 | Explain how quantitative variables were handled in the analyses. If applicable, describe which groupings were chosen and why. Basic statistic analysis was done, calculating mean and range of quantitative variables. |
| Statistical methods | 12 | (*a*) Describe all statistical methods, including those used to control for confounding |
|  |  | (*b*) Describe any methods used to examine subgroups and interactions |
|  |  | (*c*) Explain how missing data were addressed |
|  |  | (*d*) If applicable, explain how loss to follow-up was addressed |
|  |  | (*e*) Describe any sensitivity analyses  * Not applicable. It’s a descriptive study of 4 patients |
| Results | | |
| Participants | 13* | (a) Report numbers of individuals at each stage of study—eg numbers potentially eligible, examined for eligibility, confirmed eligible, included in the study, completing follow-up, and analysed  Lines 122 to 124: “From 2006 to 2016, 218 adult patients diagnosed with sporotrichosis, by fungal isolation in culture, were treated with cryosurgery. From these 218 patients, 8 were pregnant women, and 4 of them were treated exclusively with cryosurgery.” |
|  |  | (b) Give reasons for non-participation at each stage. Not applicable. |
|  |  | (c) Consider use of a flow diagram. Not applicable. |
| Descriptive data | 14* | (a) Give characteristics of study participants (eg demographic, clinical, social) and information on exposures and potential confounders  Lines 124 to 127 “These 4 patients were at the second trimester of pregnancy and their age ranged from 18 to 34 years. All of them lived in Rio de Janeiro state, Brazil. Two of them worked with domestic duties. The patients presented ulcerovegetative or nodular ulcerovegetative lesions” |
|  |  | (b) Indicate number of participants with missing data for each variable of interest  Not applicable. |
|  |  | (c) Summarise follow-up time (eg, average and total amount)  These data are summarized in Table 1 (line 140). Each cryosurgery session correspond to one month of follow-up time. |
| Outcome data | 15* | Report numbers of outcome events or summary measures over time  These data are summarized in Table 1 (line 140). “The number of cryosurgery sessions ranged from 1 to 3. All the patients were discharged after a complete cure. No adverse reactions were observed during the treatment as well as no relapses were documented after delivery.” |
| Main results | 16 | (*a*) Give unadjusted estimates and, if applicable, confounder-adjusted estimates and their precision (eg, 95% confidence interval). Make clear which confounders were adjusted for and why they were included.  Not applicable. |
|  |  | (*b*) Report category boundaries when continuous variables were categorized  Not applicable. |
|  |  | (*c*) If relevant, consider translating estimates of relative risk into absolute risk for a meaningful time period.  Not applicable. |
| Other analyses | 17 | Report other analyses done—eg analyses of subgroups and interactions, and sensitivity analyses.  Not applicable. |
| Discussion | | |
| Key results | 18 | Summarise key results with reference to study objectives  Lines 163 to 170: “All patients herein reported came from hyperendemic areas of sporotrichosis in Rio de Janeiro state, and become infected during pregnancy. None referred prior trauma with plants, but only contact and/or trauma with cats, in agreement with the zoonotic epidemic profile reported in the literature [2]. Although S. brasiliensis, could be identified in only two cases, it is well known that it is the main species involved in Rio de Janeiro epidemic. All patients presented cutaneous-limited clinical forms on the extremities, similar to previous publications [3, 5], in contrast with other mycoses, which can be more aggressive during pregnancy [15].” |
| Limitations | 19 | Discuss limitations of the study, taking into account sources of potential bias or imprecision. Discuss both direction and magnitude of any potential bias.  Limitation is inherent to the type of study and small casuistic studied. |
| Interpretation | 20 | Give a cautious overall interpretation of results considering objectives, limitations, multiplicities of analyses, results from similar studies, and other relevant evidence.  Lines 155 to 162 – Compiled results data were interpreted and compared with current scientific information published to conclude and suggest improvements in the subject as well as in patients’ healthcare.  “ Cryosurgery has been used as an adjuvant treatment in sporotrichosis, especially in residual lesions or in cases of ulcerovegetative or nodular ulcerovegetative thick lesions since it allows a good penetration of liquid nitrogen in spray form [13]. In other subcutaneous mycoses such as chromoblastomycosis, cryosurgery has been indicated as an isolated method or associated to systemic antifungal agents with good results [14]. Until now, cryosurgery for sporotrichosis treatment has been poorly explored and documented especially considering cases that involve a supposed more virulent phylogenetic species such as S. brasiliensis.”  Lines 170 to 172:  “ This work suggests that cryosurgery is a safe and well-tolerated method, easy to perform, being a promising alternative in the treatment of cutaneous sporotrichosis in pregnant women.” |
| Generalisability | 21 | Discuss the generalisability (external validity) of the study results.  It is a descriptive study about subject scarcely reported in the literature, and therefore clinical trials are needed to confirm these preliminary results. |
| Other information | | |
| Funding | 22 | Give the source of funding and the role of the funders for the present study and, if applicable, for the original study on which the present article is based.  No funding sources. |

*Give information separately for exposed and unexposed groups.

**Note:** An Explanation and Elaboration article discusses each checklist item and gives methodological background and published examples of transparent reporting. The STROBE checklist is best used in conjunction with this article (freely available on the Web sites of PLoS Medicine at http://www.plosmedicine.org/, Annals of Internal Medicine at http://www.annals.org/, and Epidemiology at http://www.epidem.com/). Information on the STROBE Initiative is available at http://www.strobe-statement.org.
